# Palmdelphin promotes myoblast differentiation and muscle regeneration

**DOI:** 10.1038/srep41608

**Published:** 2017-02-02

**Authors:** Yaping Nie, Hu Chen, Cilin Guo, Zhuning Yuan, Xingyu Zhou, Ying Zhang, Xumeng Zhang, Delin Mo, Yaosheng Chen

**Affiliations:** 1 State Key Laboratory of Biocontrol, School of Life Sciences, Sun Yat-sen University, Guangzhou, Guangdong 510006, P.R. China

## Abstract

Differentiation of myoblasts is essential in the development and regeneration of skeletal muscles to form multinucleated, contractile muscle fibers. However, the process of myoblast differentiation in mammals is complicated and requires to be further investigated. In this study, we found Palmdelphin (Palmd), a cytosolic protein, promotes myoblast differentiation. Palmd is predominantly expressed in the cytosol of myoblasts and is gradually up-regulated after differentiation. Knockdown of Palmd by small interfering RNA (siRNA) in C2C12 markedly inhibits myogenic differentiation, suggesting a specific role of Palmd in the morphological changes of myoblast differentiation program. Overexpression of Palmd in C2C12 enhances myogenic differentiation. Remarkably, inhibition of Palmd results in impaired myotube formation during muscle regeneration after injury. These findings reveal a new cytosolic protein that promotes mammalian myoblast differentiation and provide new insights into the molecular regulation of muscle formation.

Skeletal muscle is a heterogeneous and highly complex tissue having multiple functions in the vertebrate body^1^. The embryonic development, regeneration and function of skeletal muscle are dependent upon highly coordinated expression of many genes, among which MRFs (Myf5, Myogenin, MyoD, Mrf4) are vital muscle specific transcription factors^2^,^3^. During embryonic development, transcription factors Myf5, Mrf4, and MyoD are responsible for the specification of progenitor cells to become muscle lineage-committed myoblasts, whereas Myogenin, MyoD, and Mrf4 regulate the differentiation of myoblasts^4^. These and other transcription factors such as Pax7 and SIX coordinate to regulate the process of muscle differentiation, including cell cycle withdrawal, expression of muscle-specific proteins, myoblast fusion and elongation into multinucleated myofibers^5^,^6^,^7^,^8^,^9^. Even though transcription regulation is the key process in muscle differentiation, the procedure of fusion and elongation of myoblasts to form myofibers is also controlled by a multitude of membrane and cytosolic proteins, including muscle-specific membrane protein Myomaker^10^,^11^, Wnts^12^, MAPKs^13^,^14^,^15^,^16^, ELMO^17^, Brain-specific angiogenesis inhibitor 1 (BAI1)^17^, small heat shock proteins (sHSPs)^18^ and others^19^,^20^.

Mature skeletal muscle, a renewing organ, has remarkable regenerative capacity, since many satellite cells locate in the surface of myofibers^9^. The satellite cells are responsible for generating myoblasts when muscle regeneration begins after injury^21^. There are many similarities between embryonic muscle development and mature muscle regeneration, such as same transcription factors and signaling molecules^22^,^23^,^24^. Thus studies in adult muscle regeneration could provide better understandings about embryonic muscle development. The snake peptide cardiotoxin (CTX) injection can cause muscle injury and is an excellent experimental model to study the regeneration of skeletal muscle in a controlled and reproducible way^25^.

Palmd is a predominant cytosolic isoform of the Paralemmin family, which are lipid raft-associated proteins implicated in plasma membrane dynamics and cell shape control^26^,^27^. In human, *Palmd* mRNA was ubiquitously expressed, enriched in testis, prostate, heart and skeletal muscle^28^. In mice, Palmd was expressed in variable abundances in nearly all tissues, with highest expression in the heart and lung^26^. Immunofluorescence microscopy showed a punctate distribution of Palmd throughout the cytoplasm^27^. In our previous RNA-seq analysis, we found that *Palmd* expression was gradually up-regulated during embryonic muscle development in three pig strains (data not shown), indicating it might play a role in mammalian muscle development. However, the molecular and functional nature of Palmd in muscle development is unknown.

In the current study, we first found that Palmd was gradually increased during C2C12 myoblast differentiation, and activated after muscle injury. Then gain- and loss-of-function approach proved that Palmd promoted C2C12 myoblast differentiation. In addition, knockdown of Palmd expression *in vivo* resulted in impaired myotube formation after CTX injury. These results demonstrate that Palmd promote myoblast differentiation and muscle regeneration.

## Results

### Palmd expression is elevated during myoblast differentiation

**Figure 1:**
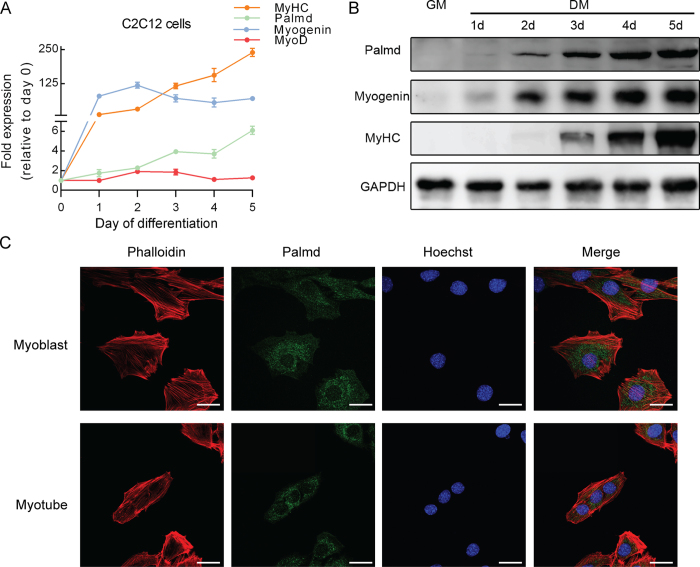
Palmd expression and location during C2C12 myoblast differentiation. (**A**) qPCR for the *Palmd, Myogenin, MyoD* and *MyHC* expression profile during C2C12 myoblast differentiation at the indicated time points, showed up-regulation of Palmd after differentiation. Data are presented as mean ± s.e.m., n = 3 per group. (**B**) The protein levels of Palmd, Myogenin, MyHC during C2C12 myoblast differentiation showed a similar result. GM: growth medium, DM: differentiation medium. (**C**) Immunofluorescence staining for Palmd (green) together with Phalloidin (red) and nucleus (blue) staining in myoblast and myotube. Scale bar = 25 μm.

To investigate the expression pattern of Palmd during myoblast differentiation, C2C12 cells were cultivated and collected at six time points of differentiation, including 0d (before induced to differentiation), 1d, 2d, 3d, 4d, 5d. Similar to the expression of Myogenin and MyHC, both the mRNA and protein level of Palmd were gradually increased during myoblast differentiation. *Palmd* mRNA was at a low level before differentiation, and increased during differentiation (Fig. 1A). Palmd protein expression was very low before differentiation, and it gradually improved during differentiation, accompanied with the increased expression of Myogenin and MyHC (Fig. 1B).

To analyze the cellular distribution of Palmd, C2C12 cells were fixed and co-stained for Phalloidin and nucleus. The results showed a discontinuous distribution of Palmd immunoreactivity, in the form of spots scattered in the cytoplasm of myoblasts and myotubes (Fig. 1C). Its cellular distribution pattern in C2C12 cells was in coincidence with previous study in NS20Y and PC12 cells^27^.

These results demonstrate that Palmd expression is increased during myoblast differentiation, indicating it may play an important role in myoblast differentiation.

### Palmd is activated in TA muscle after injury

**Figure 2:**
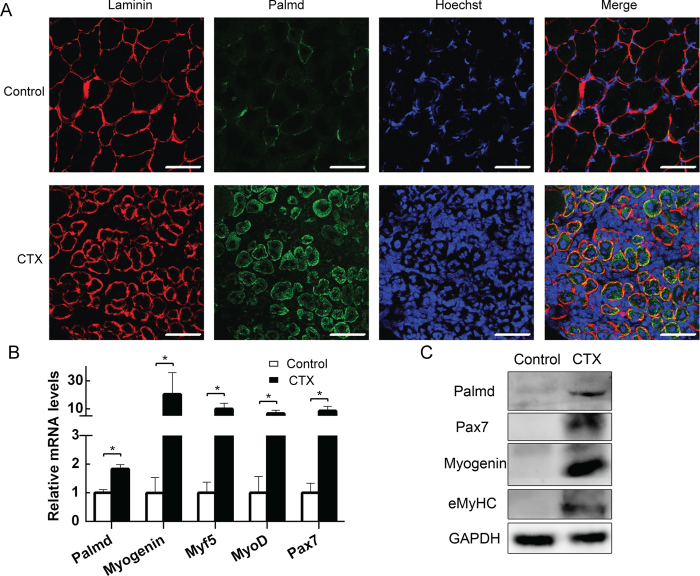
Palmd is activated in injured TA muscle. (**A**) Immunofluorescence staining for Palmd (green), Laminin (red) and nucleus (blue) staining in TA muscle sections 3.5 days after CTX or saline (control) injection. Part of Palmd (green) and Laminin (red) was merged (yellow). Scale bar = 75 μm. (**B**) The mRNA levels of *Palmd, Myogenin, Myf5, MyoD* and *Pax7* in TA muscles 3.5 days after CTX or saline injection. Data are presented as mean ± s.e.m, n = 3 per group. * p < 0.05. (**C**). The protein levels of Palmd, Myogenin, MyoD and Pax7 in TA muscles 3.5 days after CTX or saline injection.

To further explore the expression pattern of Palmd *in vivo*, tibialis anterior (TA) muscles were injured by CTX injection. TA muscle frozen section was performed on 3.5 day after injury when fibers started to form. Interestingly, the immunofluorescence results showed that Palmd was weakly expressed in healthy adult mouse TA muscle, but was highly expressed in injured regenerating TA muscle. In addition, Palmd was mostly distributed in the cytoplasm of regenerating myofibers, and part of it was located on peripheral membranes (Fig. 2A). This observation was in line with the recruitment/extraction results and the subcellular localization seen by immunofluorescence microscopy and immune-Electron microscopy, which suggested a peripheral association of Palmd with cytoskeleton-linked protein structures, or with lipid rafts or raft-enriched intrinsic membrane proteins^27^. The mRNA level of *Palmd, Myogenin, Myf5, MyoD* and *Pax7* were significantly up-regulated on 3.5d after injury (Fig. 2B). Western blotting results revealed the up-regulation of Palmd, Pax7, Myogenin and embryonic MyHC (eMyHC) expression (Fig. 2C). Together, these results prove that Palmd is activated upon injury when muscle regeneration begins, suggesting it may be essential in muscle regeneration.

### Overexpression of Palmd enhances myogenic differentiation

**Figure 3:**
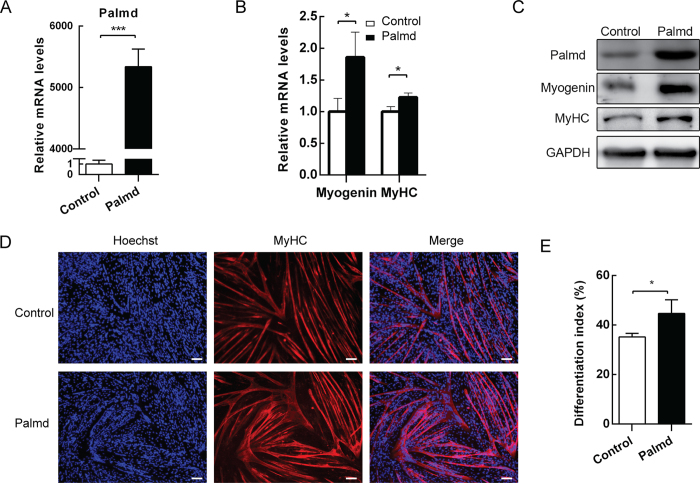
Palmd overexpression promotes C2C12 myoblast differentiation. C2C12 cells were transfected with pcDNA3.1 vector (control) or pcDNA3.1-Palmd vector (Palmd), then induced to differentiate 2d later. (**A**) The mRNA levels of *Palmd* 2d after differentiation in two groups above. (**B**) The mRNA levels of *Myogenin* 12 h after differentiation, and *MyHC* 3d after differentiation, in two groups above. (**C**) The protein levels of Palmd, Myogenin and MyHC at the time points described in (**A**) and (**B**). (**D**) After transfection and differentiation for 3 days, MyHC was detected by immunofluorescence staining. Scale bar = 100 μm. (**E**) The differentiation index in (**D**) was calculated. 3 random microscopic fields were selected for each group. Data are presented as mean ± s.e.m., n = 3 per group. *p < 0.05, ***p < 0.001.

During myoblast differentiation, the expression of Palmd was increased. To determine the role of Palmd in this process, pcDNA3.1-Palmd was transiently transfected into C2C12 cells. Palmd expression was up-regulated significantly both at mRNA level and protein level (Fig. 3A,C), which significantly increased both the mRNA and protein levels of Myogenin and MyHC (Fig. 3B and C). Myotube formation was analyzed on 3d after differentiation. Immunofluorescence assay showed that overexpression of Palmd in C2C12 cells promoted MyHC expression and generated larger myotubes with more nuclei, compared with control group (Fig. 3D). Differentiation index, a morphological parameter of myogenic differentiation, was also employed to evaluate the role of Palmd on myoblast differentiation. The result showed that overexpression of Palmd significantly elevated the differentiation index in C2C12 cells after differentiation for 3 days (Fig. 3E). Collectively, these results indicate that Palmd promotes myotube formation by enhancing myogenic commitment and differentiation.

### Knockdown of Palmd impairs myogenic differentiation

**Figure 4:**
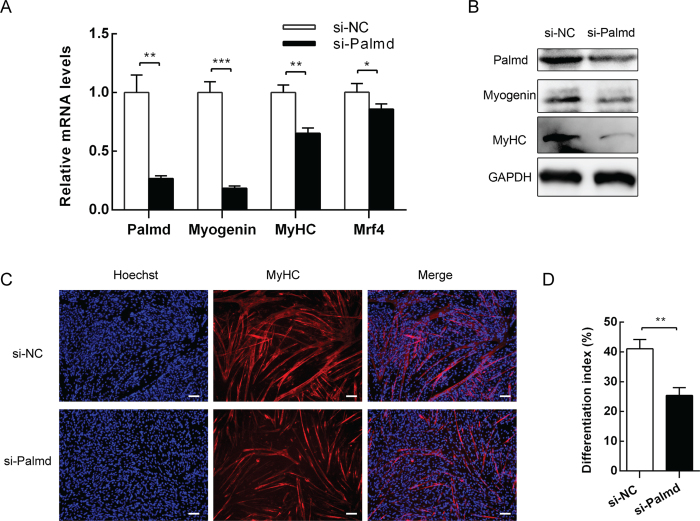
Knockdown of Palmd attenuates C2C12 myoblast differentiation. C2C12 cells were transfected with negative control siRNA (si-NC) pool or Palmd siRNA pool (si-Palmd), then induced to differentiate 2d later. (**A**) The mRNA levels of *Palmd* 2d after differentiation, and *Myogenin* 12 h after differentiation, and *MyHC, Mrf4* 3d after differentiation, in two groups above. (**B**) The protein levels of Palmd, Myogenin and MyHC at the time points described in (**A**). (**C**) After transfection and differentiation for 3 days, MyHC was detected by immunofluorescence staining. Scale bar = 100 μm. (**D**) The differentiation index in (**C**) was calculated. 3 random microscopic fields were selected for each group. Data are presented as mean ± s.e.m., n = 3 per group. *p < 0.05, **p < 0.01, ***p < 0.001.

To further investigate the role of Palmd in myoblast differentiation, endogenous Palmd was knocked down by siRNA (Fig. 4A and B). The results showed that knockdown of Palmd significantly reduced the mRNA levels of *Myogenin, MyHC*, and *Mrf4* (Fig. 4A). Additionally, the protein expression of Myogenin and MyHC were decreased (Fig. 4B). Immunofluorescence assay showed that knockdown of Palmd in C2C12 cells inhibited MyHC expression and resulted in thinner myotubes with fewer nuclei (Fig. 4C). The differentiation index was markedly declined (Fig. 4D). Therefore, these results indicate that knockdown of Palmd impairs myogenic differentiation.

### Knockdown of Palmd attenuates muscle regeneration

**Figure 5:**
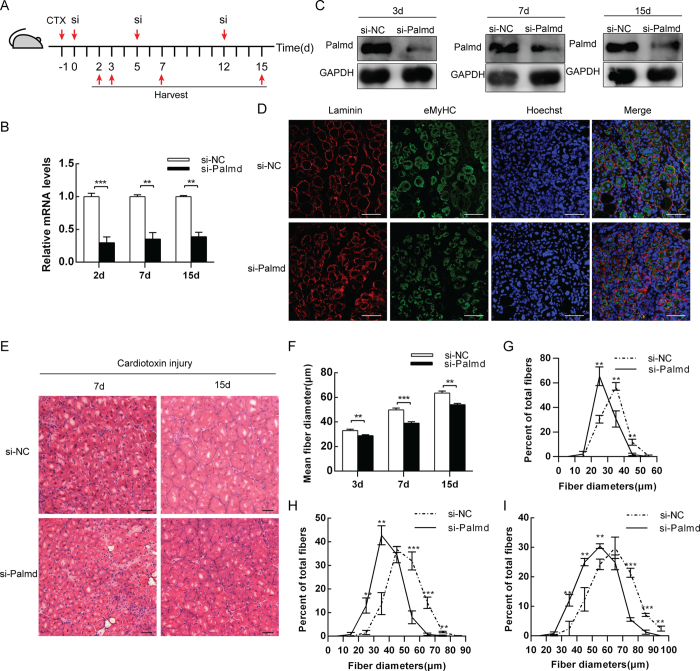
Knockdown of Palmd *in vivo* impairs muscle regeneration. (**A**) Schematic representation of CTX injury followed by si-NC or si-Palmd treatment in mouse TA muscle. (**B**) The mRNA level changes of *Palmd* in TA muscle at 2d, 7d and 15d, after CTX injury (−1d) and siRNA injection (0d, 5d and 12d). (**C**) The protein levels of Palmd in TA muscle of si-NC or si-Palmd group on 3d 7d and 15d. (**D**) Immunofluorescence staining for Laminin (red), eMyHC (green) and nucleus (blue) on si-NC or si-Palmd group TA muscle section on 3d. Scale bar = 75 μm. (**E**) H&E staining on si-NC or si-Palmd treated TA muscle section at 7d and 15d. Scale bar = 50 μm. (**F**) TA muscle average myofiber diameters (μm) of si-NC or si-Palmd group at 3d, 7d and 15d. n = 3 per group, more than 300 myofibers per mouse. (**G**~**I**) Percent distributions by diameter (μm) of myofibres at 3d (**G**), 7d (**H**) and 15d (**I**) analyzed in (**F**). Data are presented as mean ± s.e.m., n = 3 per group. **p < 0.01, ***p < 0.001.

Palmd expression was elevated in TA muscle after injury, indicating it may play a role in muscle regeneration. To find out whether Palmd can influence muscle regeneration, TA muscles of mice were injured by CTX injection and transfected with Palmd siRNA *in vivo* on the next day (0d), 5d and 12d to maintain long-term knockdown efficiency, then harvested to perform qPCR at 2d, 7d and 15d, Western blotting and frozen section at 3d, 7d and 15d during muscle regeneration (Fig. 5A). The mRNA levels of *Palmd* were decreased significantly on 2d, 7d and 15d (Fig. 5B), accompanied with decrease of its protein level (Fig. 5C). Immunofluorescence of TA sections at 3d during regeneration showed that knockdown of Palmd generated smaller myofibers (Fig. 5D), and H&E staining of TA sections at 7d and 15d during regeneration achieved similar results (Fig. 5E). The mean myofiber diameters were significantly reduced (Fig. 5F), and the distributions of myofiber diameters were shifted towards smaller diameters compared to controls (Fig. 5G,H and I). These results demonstrated that knockdown of Palmd can attenuate mouse TA muscle regeneration caused by CTX injury. Therefore, Palmd plays an essential role in muscle regeneration.

## Discussion

Skeletal muscle, which is composed of contractile multinuclear muscle fibers, is distributed in the entire vertebrate body and plays key roles in maintaining daily activities and exercise. The differentiation of muscle fibers is a complex process and regulated by a large number of genes. Transcription factors, such as MRFs, have been widely studied in myoblast differentiation. Besides, lots of membrane and cytosolic proteins participate in myoblast differentiation. In this study, we found that as a predominant cytosolic protein, Palmd could promote myoblast differentiation and muscle regeneration.

Palmd is a newly identified homolog of Paralemmin, while its function and molecular mechanism are unclear in cells. Paralemmins anchor to the cytoplasm membrane through CAAX motif at C-terminal by palmytoylation and prenylation^28^,^29^,^30^. In most Palmd mRNAs, the CAAX motif is replaced with a KKVI motif, which is the reason why Palmd is located predominantly in the cytosol. However, a single human Palmd cDNA containing a CAAX motif has been found^26^. Hence, there are two forms of Palmd, one form exists in the cytoplasm, the other form links to the membrane. The immunofluorescence assay of sections of injured regenerating TA muscle fibers confirmed this theory, for Palmd was observed to distribute both in the cytoplasm and on peripheral membranes (Fig. 2A). In the current study, we focused on the function of the cytosolic Palmd, the mouse Palmd splicing variant containing the KKVI motif was clone. It has been reported by Dashzeveg *et al*. that in human U2OS cells, in response to DNA damage, Palmd was induced by p53 with Ser46 phosphorylation, and moves into the nucleus from the cytoplasm to trigger apoptosis and control cell death^31^. However, we didn’t observe Palmd expression in the nucleus of both C2C12 cells and regenerating TA muscle fibers. This may due to the fact that both of C2C12 cells and regenerating TA muscle fibers were under normal proliferation and differentiation conditions. In addition, they found that knocking down of Palmd caused necroptosis-like cell death in response to DNA damage^31^. Given that when myoblast fusion happens during differentiation, a fraction of myoblasts within the population undergoes apoptosis^17^, we examined cell apoptosis and necrosis by double staining with propidium iodide (PI) and annexin V (AnV) analysis after C2C12 cells were induced to differentiate. However, we didn’t find significant differences on the percentage of both apoptotic cells (PI^−^AnV^+^) and necrotic cells (PI^+^) in Palmd overexpressed or knockdown C2C12 cells (Fig. S2). This is probably because in the process of normal proliferation and differentiation of C2C12 cells, Palmd does not move into the nucleus, thus it has little influence on cell death. Therefore, Palmd might regulate myoblast differentiation through other cellular events but not cell apoptosis.

In present study, we first found Palmd expression was at a very low level but not absent in myoblasts, and then gradually increased during myoblast differentiation (Fig. 1A and B). Gain- and loss-of-function method revealed that Palmd promoted myoblast differentiation, for it promoted the expression of Myogenin (a marker for early differentiation) and MyHC (a marker for terminal differentiation). Palmd was barely expressed in healthy adult muscles, but its expression increased during regeneration after injury (Fig. 2). Defective myoblast regeneration due to Palmd knockdown was confirmed *in vivo*, as indicated by smaller myofibers. Therefore, Palmd upregulation during myoblast differentiation promotes efficient differentiation, eventually leading to optimal muscle growth.

Taken together, the results presented here demonstrate that cytosolic protein Palmd can promote myoblast differentiation, and is required for optimal muscle regeneration after CTX injection. This finding would enrich the theoretical basis for muscle-associated diseases therapy. However, the underlying molecular mechanisms and cellular functions of Palmd in regulating myoblast differentiation are currently not clear and remain to be further investigated.

## Materials and Methods

### Mice

8–12 week aged female mice (C57BL/6) of SPF grade were purchased from Guangdong Medical Laboratory Animal Center and housed in the SPF condition during the experiment. All experimental procedures involving mice in this study were approved by the Animal Care and Use Committee of Guangdong Province and carried out in accordance with ethical standards.

### Cell culture

Mouse C2C12 cell line was purchased from ATCC. C2C12 cells were cultured in high-glucose DMEM with 10% (v/v) fetal bovine serum (growth medium) and were maintained at 37 °C in 5% CO2 incubator. Culture medium was changed to DMEM with 2% horse serum (differentiation medium) to induce differentiation when cells reached confluence.

### Plasmids, siRNAs and transfection

For Palmd expression vector, mouse Palmd was amplified by PCR using specific primers (listed in Table S1) and inserted into *KpnI* and *XhoI* sites in pcDNA3.1 vector (Invitrogen, Shanghai, China). Palmd siRNAs (listed in Table S2) and negative control siRNAs were purchased from Dharmacon (USA). C2C12 cells were transfected with plasmids or siRNAs using Lipofectamine 2000 (Invitrogen) according to the manufacturer’s instruction. All transfections were performed in triplicate for each experiment.

### RNA isolation and Quantitative real-time PCR

Total RNA was extracted from either mouse tissue or cultured cells with TRIZOL (Invitrogen) and cDNA was synthesized from 1 μg total RNA by Reverse Transcription Kit (Promega, Shanghai, China). Quantitative real-time PCR (qPCR) assays were performed on the LightCycler 480 system (Roche, Basel, Switzerland) using SYBR Green qPCR Mix (Dongsheng Biotech, Guangzhou, China), with GAPDH as an internal control for normalization. Primer sequences to detect gene expression are listed in Table S1.

### Western Blotting

Protein extracts were obtained following collection of cultured C2C12 cells or mouse TA muscles in lysis buffer (50 mM Tris, 150 mM NaCl, 1% Triton X-100, 0.1% SDS, 1% Sodium deoxycholate, pH 8.0, and freshly added protease inhibitor PMSF), then electrophoresed on 6% or 9% (w/v) SDS-PAGE and transferred to 0.45 μm PVDF membrane (Roche). The membranes were blocked with 3% BSA for 1 h and then incubated with primary antibodies at 4 °C overnight, followed by incubation with proper secondary antibodies. Blots were visualized using a commercial enhanced chemiluminescene (ECL) detection kit (Thermo Scientific, Beijing, China). Primary antibodies used included Myog (#MAB3876, Millipore), MyHC (#ab7784, Abcam), GAPDH (#2118 S, CST), Pax7 (DSHB) Palmd (customized in Anbang biotechnology, Guangzhou, China. Validation information of this antibody is shown in Fig. S1 and Table S3). Secondary antibodies used were either anti-rabbit HRP-linked (#7074 S, CST) or anti-mouse HRP-linked (#7076 S, CST) antibodies.

### CTX injury

CTX (Sigma, Shanghai, China) was dissolved in sterile saline to a final concentration of 10 mM. Mouse legs were cleaned with alcohol. Right TA muscles of mice (C57BL/6) were injected through the skin with 50 μl of CTX by a hypodermic syringe. Left TA muscles were injected with sterile saline only.

### Mouse TA muscle transfection

Reagent A was prepared by mixing 12.5 μg siRNA (si-NC or si-Palmd, 1 μg/ul) with 12.5 μl sterile saline. Reagent B was prepared by mixing 6.25 μl Entranster™-*in vivo* (Engreen, Beijing, China) with 18.75 μl sterile saline. Reagent B was then added to A and mixed thoroughly to form mixture. After a 15 minute incubation period at room temperature, the mixture was injected into mouse TA muscles through the skin by a hypodermic syringe.

### H&E staining of frozen sections

The mouse TA muscle frozen blocks for section were prepared following a video article on Jove^29^. Then, frozen blocks embedded in O.C.T. compound (Optimal Cutting Temperature compound) were sectioned at 10 μm using Cryostat Microtome. Frozen TA muscle sections were stained using H&E staining kit (Jiancheng Biotechnology, Nanjing, China) according to the manufacturer’s instruction.

### Immunofluorescence assay

Differentiated myotubes were fixed with 4% PFA/PBS, permeabilized with 0.5% Triton X-100 in PBS, blocked with 3% BSA/PBS, incubated with primary antibodies at 4 °C overnight, then incubated with proper secondary antibodies for 2 hours.

After being fixed with cold acetone, TA muscle section immunofluorescence was performed using Mouse on Mouse Polymer IHC Kit (Abcam) according to the manufacturer’s instruction.

Observation and images were captured by fluorescent reverse microscopy (ZEISS, Heidenheim, Germany) and confocal microscopy (Leica Microsystems, Deerfield, IL). Primary antibodies used included Palmd (customized in Anbang biotechnology, Guangzhou, China), MyHC (#ab7784, Abcam), Laminin (#ab11576, Abcam), embryonic MyHC (#F1.652, DSHB). Secondary antibodies labeled with FITC 488, 555 or 647 fluorochrome (CST) were used.

### Flow cytometry analysis

C2C12 cells were transfected with siRNA (si-NC or si-Palmd) or overexpression plasmids (pcDNA3.1 or pcDNA3.1-Palmd). 48 hours later, cells were induced to differentiate and incubated for 12 h. Cells were digested with trypsin and washed by PBS for 2 times, then resuspended and stained with propidium iodide (PI) and annexin V (AnV) using Annexin V-FITC Apoptosis Detection Kit (Sigma) according to the manufacturer’s instruction. After a 10 minute incubation period at room temperature, the cells are analyzed by BD FACSCalibur system (BD Biosciences, Franklin Lakes, USA).

### Quantification and statistics

The differentiation index was calculated as the percentage of nuclei in MyHC-positive cells that contained at least 2 nuclei. Data are presented as mean ± s.e.m from three independent experiments. Differences between groups were tested for statistical significance using an unpaired two-tailed Student’s t-test. P < 0.05 was considered as significance.

## Additional Information

**How to cite this article**: Nie, Y. *et al*. Palmdelphin promotes myoblast differentiation and muscle regeneration. *Sci. Rep.* **7**, 41608; doi: 10.1038/srep41608 (2017).

**Publisher's note:** Springer Nature remains neutral with regard to jurisdictional claims in published maps and institutional affiliations.

## Supplementary Material

Supplementary Information

## References

[b1] Baskin K. K., Winders B. R. & Olson E. N. Muscle as a “mediator” of systemic metabolism. Cell Metab. 21, 237–48 (2015). 25651178 10.1016/j.cmet.2014.12.021 PMC4398026

[b2] Apponi L. H., Corbett A. H. & Pavlath G. K. RNA-binding proteins and gene regulation in myogenesis. Trends Pharmacol Sci. 32, 652–8 (2011). 21982546 10.1016/j.tips.2011.06.004 PMC3214663

[b3] Berkes C. A. & Tapscott S. J. MyoD and the transcriptional control of myogenesis. Semin Cell Dev Biol. 16, 585–95 (2005). 16099183 10.1016/j.semcdb.2005.07.006

[b4] Tajbakhsh S. Skeletal muscle stem and progenitor cells: reconciling genetics and lineage. Exp Cell Res. 306, 364–372 (2005). 15882864 10.1016/j.yexcr.2005.03.033

[b5] Bentzinger C. F., Wang Y. X. & Rudnicki M. A. Building muscle: molecular regulation of myogenesis. Cold Spring Harb Perspect Biol. 1, 4(2) (2012). 10.1101/cshperspect.a008342 PMC3281568 22300977

[b6] Liu Y., Chu A., Chakroun I., Islam U. & Blais A. Cooperation between myogenic regulatory factors and SIX family transcription factors is important for myoblast differentiation. Nucleic Acids Res. 38, 6857–6871 (2010). 20601407 10.1093/nar/gkq585 PMC2978361

[b7] Braun T. & Gautel M. Transcriptional mechanisms regulating skeletal muscle differentiation, growth and homeostasis. Nat Rev Mol Cell Biol. 12, 349–361 (2011). 21602905 10.1038/nrm3118

[b8] Bryson-Richardson R. J. & Currie P. D. The genetics of vertebrate myogenesis. Nat Rev Genet. 9, 632–646 (2008). 18636072 10.1038/nrg2369

[b9] Dumont N. A., Wang Y. X. & Rudnicki M. A. Intrinsic and extrinsic mechanisms regulating satellite cell function. Development. 142, 1572–1581 (2015). 25922523 10.1242/dev.114223 PMC4419274

[b10] Millay D. P. . Myomaker is a membrane activator of myoblast fusion and muscle formation. Nature. 499, 301–305 (2013). 23868259 10.1038/nature12343 PMC3739301

[b11] Millay D. P., Sutherland L. B., Bassel-Duby R. & Olson E. N. Myomaker is essential for muscle regeneration. Genes Dev. 28, 1641–1646 (2014). 25085416 10.1101/gad.247205.114 PMC4117939

[b12] von Maltzahn J., Chang N. C., Bentzinger C. F. & Rudnicki M. A. Wnt signaling in myogenesis. Trends Cell Biol. 22, 602–609 (2012). 22944199 10.1016/j.tcb.2012.07.008 PMC3479319

[b13] Jones, N. C., Fedorov Rosenthal Y. V., Olwin R. S, ERK1/2 is required for myoblast proliferation but is dispensable for muscle gene expression and cell fusion. J Cell Physiol. 186, 104–15 (2001). 11147804 10.1002/1097-4652(200101)186:1<104::AID-JCP1015>3.0.CO;2-0

[b14] Z. Wu . p38 and extracellular signal-regulated kinases regulate the myogenic program at multiple steps. Mol Cell Biol. 20, 3951–64 (2000). 10805738 10.1128/mcb.20.11.3951-3964.2000 PMC85749

[b15] Bhatnagar S., Kumar A., Makonchuk D. Y., Li H. & Kumar A. Transforming growth factor-beta-activated kinase 1 is an essential regulator of myogenic differentiation. J Biol Chem. 285, 6401–6411 (2010). 20037161 10.1074/jbc.M109.064063 PMC2825435

[b16] Segales J. . Chromatin-wide and transcriptome profiling integration uncovers p38alpha MAPK as a global regulator of skeletal muscle differentiation. Skelet Muscle. 15, 6–9 (2016). 10.1186/s13395-016-0074-x PMC4791895 26981231

[b17] Hochreiter-Hufford A. E. . Phosphatidylserine receptor BAI1 and apoptotic cells as new promoters of myoblast fusion. Nature. 497, 263–267 (2013). 23615608 10.1038/nature12135 PMC3773542

[b18] Dubinska-Magiera M. . Contribution of small heat shock proteins to muscle development and function. FEBS Lett. 588, 517–530 (2014). 24440355 10.1016/j.febslet.2014.01.005

[b19] Kim J. H., Jin P., Duan R. & Chen E. H. Mechanisms of myoblast fusion during muscle development. Curr Opin Genet Dev. 32, 162–170 (2015). 25989064 10.1016/j.gde.2015.03.006 PMC4508005

[b20] Hindi S. M., Tajrishi M. M. & Kumar A. Signaling mechanisms in mammalian myoblast fusion. Sci Signal. 6, re2 (2013). 10.1126/scisignal.2003832 PMC3724417 23612709

[b21] Relaix F. & Zammit P. S. Satellite cells are essential for skeletal muscle regeneration: the cell on the edge returns centre stage. Development. 139, 2845–2856 (2012). 22833472 10.1242/dev.069088

[b22] Tajbakhsh S. Skeletal muscle stem cells in developmental versus regenerative myogenesis. J Intern Med. 266, 372–389 (2009). 19765181 10.1111/j.1365-2796.2009.02158.x

[b23] Kang J. S. & Krauss R. S. Muscle stem cells in developmental and regenerative myogenesis. Curr Opin Clin Nutr Metab Care. 13, 243–248 (2010). 20098319 10.1097/MCO.0b013e328336ea98 PMC2872152

[b24] Yusuf F. & Brand-Saberi B. Myogenesis and muscle regeneration. Histochem Cell Biol. 138, 187–199 (2012). 22644378 10.1007/s00418-012-0972-x

[b25] Mahdy M. A., Lei H. Y., Wakamatsu J., Hosaka Y. Z. & Nishimura T. Comparative study of muscle regeneration following CTX and glycerol injury. Ann Anat. 202, 18–27 (2015). 26340019 10.1016/j.aanat.2015.07.002

[b26] Hu B., Copeland N. G., Gilbert D. J., Jenkins N. A. & Kilimann M. W. The paralemmin protein family: identification of paralemmin-2, an isoform differentially spliced to AKAP2/AKAP-KL, and of palmdelphin, a more distant cytosolic relative. Biochem Biophys Res Commun. 285, 1369–1376 (2001). 11478809 10.1006/bbrc.2001.5329

[b27] Hu B., Petrasch-Parwez E., Laue M. M. & Kilimann M. W. Molecular characterization and immunohistochemical localization of palmdelphin, a cytosolic isoform of the paralemmin protein family implicated in membrane dynamics. Eur J Cell Biol. 84, 853–866 (2005). 16323283 10.1016/j.ejcb.2005.07.002

[b28] Nuria Andreua M. E. . PALML, a novel paralemmin-related gene mapping on human chromosome 1p21. Gene. 278, 33–40 (2001). 11707320 10.1016/s0378-1119(01)00719-3

[b29] Meng H. . Tissue triage and freezing for models of skeletal muscle disease. J Vis Exp. 15, 89 (2014). 10.3791/51586 PMC4215994 25078247

[b30] Christian Kutzleb G. S. . Paralemmin, a prenyl-palmitoyl-anchored phosphoprotein abundant in neurons and implicated in plasma membrane dynamics and cell process formation. J Cell Biol. 143, 795–813 (1998). 9813098 10.1083/jcb.143.3.795 PMC2148134

[b31] Dashzeveg N., Taira N., Lu Z. G., Kimura J. & Yoshida K. Palmdelphin, a novel target of p53 with Ser46 phosphorylation, controls cell death in response to DNA damage. Cell Death Dis. 5, e1221 (2014). 24810057 10.1038/cddis.2014.176 PMC4047856

