# Supplementary material for: Palmdelphin promotes myoblast differentiation and muscle regeneration
Source: Sci Rep. 2017 Feb 2;7:41608. doi: 10.1038/srep41608 (PMC5288731; doi:10.1038/srep41608)
Supplement: Supplementary Information [file srep41608-s1.pdf]

Supplementary information

Palmdelphin promotes myoblast differentiation and muscle regeneration

Yaping Nie, Hu Chen, Cilin Guo, Zhuning Yuan, Xingyu Zhou, Ying Zhang, Xumeng Zhang, Delin Mo and Yaosheng Chen

Table S1. Primers sequences (5' to 3')

Group and name	sequence
**Primers for gene cloning**	
Palmdelphin	F: GTGGGTACCATGGAAGAAGCTGAGCTG
	R: CGCTCGAGTCAGATCACCTTTTTCCCCAGCTTG
**Primers for qPCR**	
Palmdelphin	F: ATCTCACAGAAGCGTCTGAAAAT
	R: CTGCCGATTCCATCCAGGAG
Myogenin	F: GCAATGCACTGGAGTTCG
	R: ACGATGGACGTAAGGGAGTG
MyHC	F: AGCTTGAAAACGAGGTGGAA
	R: CCTCCTCAGCCTGTCTCTTG
Myod	F: GCCTGAGCAAAGTGAATGAG
	R: GCAGACCTTCGATGTAGCG
Myf5	F: CCTGTCTGGTCCCGAAAGAAC
	R: GACGTGATCCGATCCACAATG
Pax7	F: CTGCTGAAGGACGGTCACTG
	R: GGA TGCCATCGA TGCTGTGT
Mrf4	F: CTACATTGAGCGTCTACAGGACC
	R: CTGAAGACTGCTGGAGGCTG

Table S2. siGENOME Mouse Palmdelphin (114301) siRNA-SMARTpool, 5 nmol

SmartPool content	sequence
Target sequence 1	CUACAUACCUUCCCGAUUA
Target sequence 2	GAAGUUAGGCCGUAUGAAA
Target sequence 3	UGUCAGAUAUAACAUCGUU
Target sequence 4	GCAAAACGAACACGAAGUU

Table S3. Antigen peptide sequence

Epitope	Length	AA region	Term	Purity
NH2-CSERNSKSPTEYHE-CONH2	14	246-258	Center	>90%

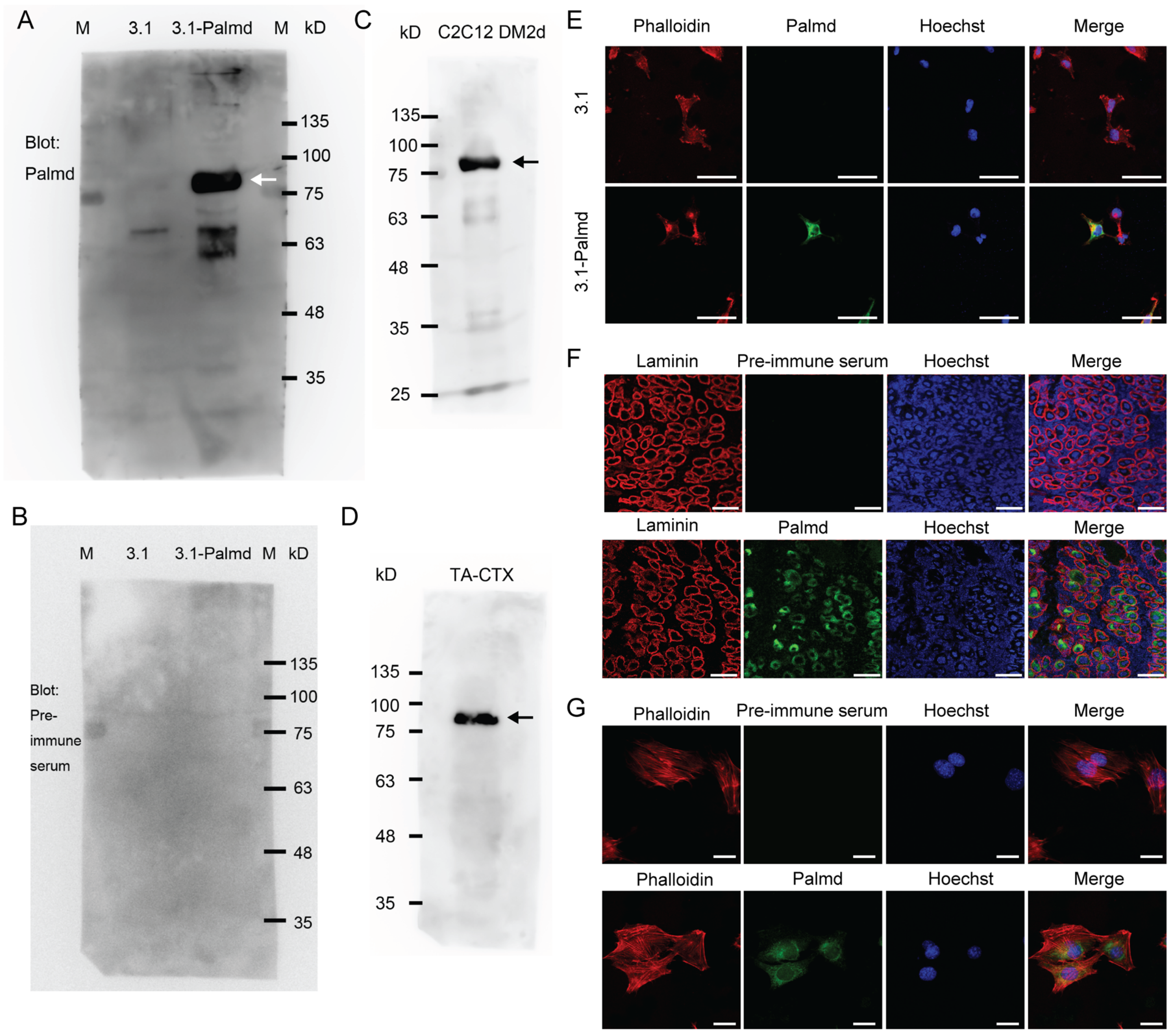

Fig. S1. WB and IF validation of the Palmd antibody used in this research. (A) 293T cells were transfected with pcDNA3.1 or pcDNA3.1-Palmd, 48 h later, cell protein extracts were collected to perform WB. Palmd antibody was used as the primary antibody. White arrow indicates the band of Palmd, between 75 kD and 100 kD. M: marker, 3.1: pcDNA3.1, 3.1-Palmd: pcDNA3.1-Palmd. (B) Palmd antibody was replaced by pre-immune serum, the negative control, to perform the same WB experiments in (A). (C) A full-length WB lane of protein lysates from C2C12 cells differentiated for 2 days. Black arrow indicates the band of Palmd. (D) A full-length WB lane of protein lysates from TA muscles 3 days after CTX injury. Black arrow indicates the band of Palmd.. (E) 293T cells were transfected with pcDNA3.1 or pcDNA3.1-Palmd, 48 h later, cells were stained with Phalloidin (red), Palmd (green) and nucleus (blue). Scale bar = 50 μm. (F) CTX injured TA muscle sections were stained for Laminin (red) and nucleus (blue), and performed immunofluorescence staining using pre-immune serum (upper group) or Palmd antibody (lower group) as the primary antibody. Scale bar = 75 μm. (G) C2C12 myoblasts were stained for Phalloidin (red) and nucleus (blue), and performed immunofluorescence staining using pre-immune serum (upper group) or Palmd antibody (lower group) as the primary antibody. Scale bar = 25 μm.

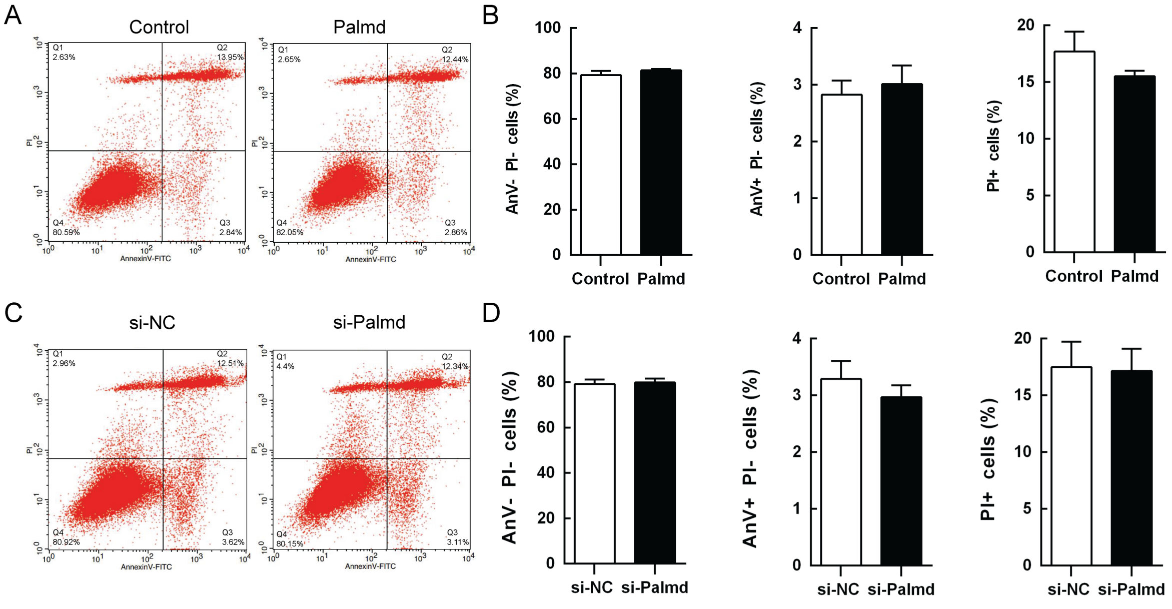

Fig. S2. Palmd does not influence cell apoptosis and necrosis during myoblast differentiation. (A) C2C12 cells were transfected with overexpression plasmids pcDNA3.1 (control) or pcDNA3.1-Palmd (Palmd). 48 hours later, cells were induced to differentiate and incubated for 12 h, followed by double staining with propidium iodide (PI) and annexin V (AnV). PI^- AnV^- populations were identified as viable cells, PI^-AnV^+ populations were defined as apoptotic cells and PI^+ populations indicated necrotic cells. (B) Percentage of viable (left panel), apoptotic (middle panel), or necrotic (right panel) cells from the data described in (A) was calculated. Data are presented as mean± s.e.m. , n = 3 per group. (C) C2C12 cells were transfected with si-NC or si-Palmd. 48 hours later, cells were induced to differentiate and incubated for 12 h, then stained with PI and AnV. (D) Percentage of viable (left panel), apoptotic (middle panel), or necrotic (right panel) cells from the data described in (C) was calculated. Data are presented as mean± s.e.m. , n = 3 per group.
